# Anti-plaque efficacy of two commercially available dentifrices in orthodontic patients. A randomized controlled clinical trial

**DOI:** 10.1186/s12903-026-08879-2

**Published:** 2026-06-19

**Authors:** Krishna Nanaware, Vinutha Bhat, Vibha Acharya, Arun Urala, Jothi Varghese

**Affiliations:** 1 Department of Periodontology, Manipal College of Dental Sciences, Manipal Academy of Higher Education, Manipal, India; 2 Department of Biochemistry, Kasturba Medical College, Manipal Academy of Higher Education, Manipal, India; 3 Department of Forensic Medicine and Toxicology, Kasturba Medical College, Manipal Academy of Higher Education, Manipal, India; 4 Department of Orthodontics and Dentofacial Orthopaedics, Manipal College of Dental Sciences, Manipal Academy of Higher Education, Manipal, India

**Keywords:** Gingivitis, Enzymatic-dentifrice, Oxidative-stress biomarkers, Orthodontic treatment

## Abstract

**Background:**

The present study compares the anti-plaque efficacy of two commercially available dentifrices containing natural salivary enzymes in patients undergoing fixed orthodontic therapy.

**Methodology:**

This prospective, parallel- arm, double-blind, randomized controlled trial included 54 participants undergoing fixed orthodontic therapy who were diagnosed with moderate to severe gingivitis and were randomly allocated into three groups (*n* = 18 per group). Group A received Dente 91^®^ dentifrice, Group B received Enzycal 950™ dentifrice, and Group C served as the control group. Intergroup differences were analysed using one-way ANOVA followed by Bonferroni post hoc test. Within-group comparisons across multiple time points were assessed using repeated measures ANOVA with post hoc analysis, while paired t-test was used for comparisons between two-time intervals. Correlations between clinical and biochemical parameters were evaluated using Pearson’s correlation test. Primary outcomes included clinical parameters- Plaque Index (PI), Gingival Index (GI), and Bleeding on probing (BOP) which were assessed at baseline, 6, 12, and 24 weeks. The secondary outcomes involved evaluation of salivary biomarkers namely Advanced Oxidative Protein Products (AOPP) and Myeloperoxidase (MPO) levels which were estimated at baseline and at 24 weeks.

**Results:**

Statistical analysis demonstrated significant differences between and within the three experimental groups at different time intervals with the level of significance set at *p* < 0.05. Both enzyme-based dentifrices (Groups A and B) showed significantly greater improvements in PI, GI, and BOP compared to the Group C (control) (*p* < 0.05). The salivary biomarkers, AOPP significantly decreased in Groups A and B (*p* = 0.0002), compared to Group C at 24th week study interval. Strong positive correlations were found between clinical indices (PI, GI, BOP) and AOPP (*p* < 0.001) whereas MPO levels did not exhibit a significant correlation (*p* = 0.76) with any of the measured clinical parameters at study time points.

**Conclusion:**

These findings suggest that enzyme-based dentifrices provide effective plaque control and may additionally contribute to reduction in oxidative stress, thereby offering dual benefits for patients undergoing orthodontic treatment. This clinical trial was prospectively registered on 21st July 2023 with the Clinical Trials Registry- India (CTRI/2023/07/055494).

## Background

Plaque-induced gingivitis, the earliest and reversible form of periodontal disease, impacts up to 90% of the global population, making it one of the most prevalent oral inflammatory conditions [1]. It primarily arises from the accumulation of biofilm-associated bacteria at the gingival margin. Although gingivitis can be managed by routine oral hygiene regimen and professional dental care, certain interventions like fixed orthodontic treatment may hinder effective plaque control measures and increase the risk of gingival inflammation.

Orthodontic appliances introduce multiple challenges to gingival health, including mechanical irritation from bands, chemical irritation from cements, food impaction near arch wires, and reduced efficacy of oral hygiene practices [2]. The close contact of these appliances with gingival tissues promotes biofilm retention, triggering local inflammatory responses and gingival overgrowth [3]. If not regularly disrupted, the biofilm can lead to an imbalance in the oral microbiota, promoting the growth of pathogenic species and contributing to persistent inflammation, alveolar bone loss, and gingival recession [4].

Effective plaque control is crucial for maintaining oral health during orthodontic treatment. While mechanical tooth brushing is the cornerstone of plaque removal, adjunctive strategies such as oral rinses and irrigators have also been recommended [5]. However, long-term adherence to such measures remains limited among the general population [6]. Incorporating antibacterial agents into dentifrices provides a practical alternative, especially for individuals struggling with consistent mechanical plaque removal [7]. Tooth brushing combined with these adjunctive formulations has been shown to improve biofilm disruption and enhance gingival health compared to standard fluoride dentifrices [8].

Enzyme-containing dentifrices, which utilize naturally occurring salivary enzymes, have emerged as promising adjuncts for biofilm control. One such formulation includes lactoferrin (LF) and nano-hydroxyapatite crystals. LF exerts antimicrobial effects via two mechanisms: chelation of iron depriving bacteria of essential nutrients and direct disruption of bacterial membranes, particularly gram-negative species, through membrane lysis and lipopolysaccharide release [9]. Another enzyme-based dentifrice features a triple enzyme system comprising of lactoperoxidase, amyloglucosidase, and glucose oxidase. This system mimics natural salivary defences by producing hypothiocyanite, which disrupts bacterial metabolism by oxidizing sulfhydryl groups in the bacterial membrane, impairing glucose transport and cellular integrity. The peroxidase reaction is supported by hydrogen peroxide generated from glucose and amyloglucosidase-mediated starch breakdown [10].

Orthodontic tooth movement initiates an inflammatory-like response involving vascular changes, leukocyte migration and release of enzymes and cytokines. Additionally, the metal appliance corrosion, elevates reactive oxygen species, with salivary AOPP and MPO serving as non-invasive oxidative stress markers reflecting protein oxidation and neutrophil-mediated tissue responses [11–14].

In two separate studies, Daly et al. [15] and Paque et al. [16] have tested the efficacy of enzymatic dentifrice on patients diagnosed with chronic periodontitis. The authors have concluded beneficial antiplaque effect. Given the increased risk of plaque accumulation and gingivitis during orthodontic therapy, a randomized clinical trial was conducted to evaluate the anti-plaque efficacy of two enzymatic dentifrices. The primary objective was to assess their effectiveness in managing chronic gingivitis, while the secondary objective was to analyze changes in salivary antioxidant levels from baseline to 24 weeks. Hence, the null hypothesis was that at the end of 24-week follow-up period, there would be no difference in the anti-plaque effect of the tested dentifrices compared to the control dentifrice.

## Methods

### Study design and sample size determination

This single-centre, prospective, double-blinded randomized controlled three-arm parallel design clinical trial was conducted on patients undergoing fixed orthodontic therapy and was referred to the Department of Periodontology. This clinical investigation was approved by the Institutional ethics committee (IEC 108/2023, dated 14th July 2023) and was conducted in accordance with the Helsinki Declaration of 1975, as revised in 2013 [17]. The present study followed the CONSORT 2010 guidelines and was a prospectively registered with the clinical trials registry- India (CTRI/2023/07/055494) on July 21st, 2023. Participants who visited the department of Periodontology, Manipal College of Dental Sciences, Manipal Academy of Higher Education, Manipal were recruited in the time frame between December 2023 and August 2024.

Participant eligibility included healthy individuals age between 18 and 30 years undergoing fixed orthodontic therapy (with 0.022-inch slot, MBT prescription, stainless steel appliance) presented with at least 24 functional teeth and exhibited moderate to severe gingivitis as per the 2017 classification [18]. Individuals with advanced periodontal disease, systemic illness, or who had received antibiotic therapy in the preceding six months were excluded.

For sample size determination, a power analysis was established by G*power, version 3.0.1(Franz Faul, Universität Kiel, Germany). Based on previous studies [15, 16] the minimum sample size of 15 patients per group was required to attain 90% power at a 5% level of significance with a 95% confidence interval (CI) and an effect size of 0.25, which was estimated from changes in plaque and gingival indices.Considering 20% dropout rate, the sample size was increased to 18 patients per group.

### Patient recruitment and trial process

**Fig. 1:**
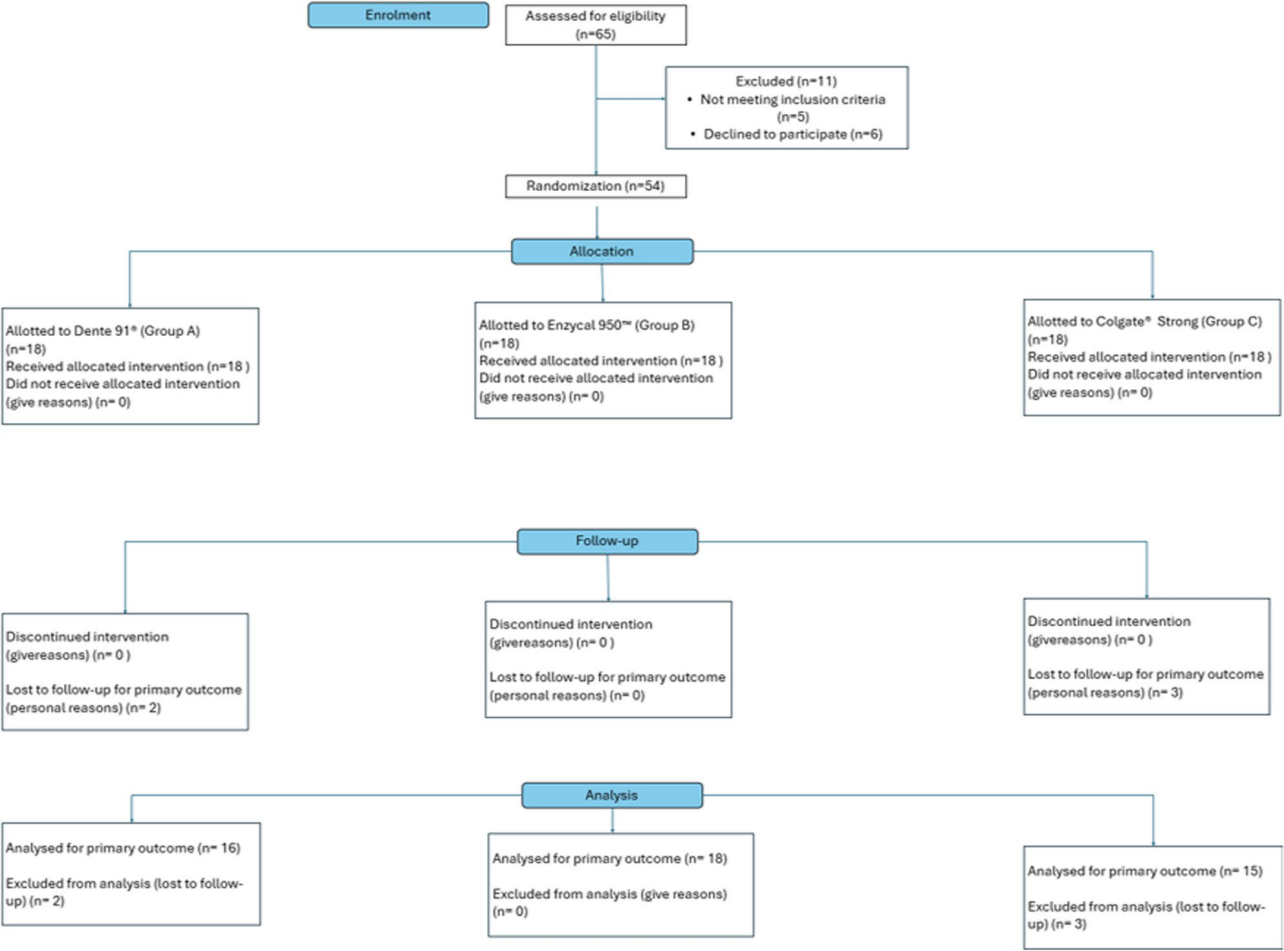
Consort flow diagram

The initial screening was conducted by the co-investigator (JV), during which 65 patients were assessed for eligibility. Subjects meeting the predefined inclusion criteria were included resulting in a final sample size of 54. Eligible subjects were informed in detail about the study protocol and clinical trial procedures, and written informed consent was obtained prior to enrolment. A CONSORT flow diagram outlining the participant selection and treatment strategy is represented in Fig. 1. Block randomization was used to allocate the participants into study groups. The randomization sequence was generated using a computer-generated random number list (www.randomizer.org/)*.* Allocation concealment was done using a sequentially numbered opaque sealed envelope (SNOSE) method with a 1:1 allocation ratio. Allocation concealment from the principal investigator (KN) was achieved using sealed, coded envelopes containing the treatment allocation. Another investigator (AU) who was not involved in data collection, managed the sealed coded envelopes and opened them after the clinical procedure was completed.

Participants were randomly allocated to one of three groups receiving the experimental dentifrices: Group A received Dente 91^®^ (containing nano-hydroxyapatite and lactoferrin), Group B received Enzycal 950™ (containing amyloglucosidase, glucose oxidase, and lactoperoxidase), and Group C (control) received Colgate^®^ Strong Teeth. To ensure blinding, all dentifrice tubes were coated with an identical plain white colour and assigned coded labels to conceal group allocation. Both patients and the principal investigator (PI) were blinded to group allocation. The PI (KN) was limited to performing the oral examination, conducting the clinical procedure, and assessing outcomes, and remained blinded to group allocation throughout the study. At the initial visit, the patients were asked to provide 5 ml of unstimulated saliva which was collected between 9:00 am and 11:00 am to minimize the influence of circadian variations in salivary flow. Participants were instructed to refrain from eating or drinking for at least 2 h prior to sample collection and to avoid tooth brushing for 1 h before the procedure. During collection, patients were seated in an upright position and asked to allow saliva to accumulate in the floor of the mouth and passively drool into sterile collection containers using the unstimulated drooling method over a period of 5 min. This procedure was performed in accordance with the method described by Navazesh M and Kumar SK [19]. The saliva samples were stored at − 80 °C until further biochemical analysis. Following which, the principal investigator (KN) recorded the clinical parameters which included the plaque index, gingival index and bleeding on probing. All the participants were recalled at the 6th, 12th and 24th week time interval, wherein all the clinical parameters were reassessed and documented. At the final follow up visit (24th week), saliva samples were again procured for post intervention biomarker evaluation.

### Clinical outcome assessment

Primary outcome included Plaque Index [20], Gingival Index [21], and Bleeding on Probing [22]. The secondary outcome comprised of biochemical evaluation of salivary Advanced Oxidation Protein Products (AOPP) and Myeloperoxidase (MPO) levels. AOPP was quantified using a method adapted from Mesquita et al. (2014), involving the reaction of protein carbonyl groups with 2,4-dinitrophenylhydrazine (DNPH) to form yellow-colored hydrazones, which were measured spectrophotometrically [23]. The method also involved a Fenton-type reaction using hydrogen peroxide and metal ions to induce oxidative stress. MPO levels were assessed using a human MPO ELISA kit (Clementia Biotech) as per the manufacturer’s instructions. The assay measured MPO activity based on the consumption of the chromophore TNB through the formation of taurine chloramine, with one unit defined as the amount of enzyme required to consume 1.0 nanomole of TNB per minute at 25 °C [24]. 

### Post care instructions

All patients were instructed in the Modified Bass tooth-brushing technique and advised using 1 cm of the assigned experimental dentifrice, brushing twice daily for 2–3 min. A standardized orthodontic V-cut toothbrush was provided to all groups to ensure uniform mechanical plaque control. Participants were instructed not to use adjunctive interdental aids during the study period to standardize plaque control measures.

### Statistical analysis

Data was analysed using the statistical package SPSS 26.0 (SPSS Inc., Chicago, IL) and level of significance was set at *P* < 0.05. Descriptive statistics were expressed as mean ± standard deviation (SD) for the respective groups. Normality of the data was assessed by Shapiro wilk test. Plaque index, gingival index, bleeding on probing (BOP), and AOPP levels demonstrated normal distribution (*p* > 0.05), whereas MPO levels deviated from normality (*p* < 0.05). Accordingly, parametric tests were applied for normally distributed variables, and non-parametric analysis was performed for MPO levels. Inferential statistics to determine the differences between the groups were performed using one-way analysis of variance (ANOVA) test followed by Bonferroni Post hoc test. Within- group comparisons across different study time intervals were analysed using repeated measures ANOVA with Post hoc test. A paired t test was also used for within-group comparisons when only two-time intervals were involved. Correlation between clinical and biochemical parameters was done with Pearson’s Correlation test. However, for salivary biomarker, MPO levels did not show normal distribution, hence intergroup comparison was performed using Kruskal -Wallis test.

## Results

A total of 54 participants (33 females, 21 males; mean age: 22.52 ± 1.06 years) were included in the study. However, only 49 patients completed the 24-week follow-up. Five participants dropped out (2 from Group A, 3 from Group C). Hence, the final group distribution included 16 patients in Group A (Dente 91^®^), 18 in Group B (Enzycal 950™), and 15 in Group C Colgate^®^ which served as control as described in Fig. 1.

Table 1 presents the mean plaque index (PI) scores at different study time intervals (baseline, 6 weeks, 12 weeks, and 24 weeks). Intergroup comparison at baseline demonstrated that all participants had moderate plaque scores, with no statistically significant difference among the groups (ANOVA, *p* = 0.20, η² = 0.03), indicating acceptable baseline comparability.

**Table 1:** Comparison of plaque index between study groups at different time intervals

		*N*	Baseline	6 weeks	12 weeks	24 weeks
GROUP A		16	1.92 ± 0.05	1.24 ± 0.44	1.11 ± 0.39	0.92 ± 0.32
GROUP B		18	1.91 ± 0.06	1.35 ± 0.20	1.18 ± 0.18	0.88 ± 0.35
GROUP C		15	1.89 ± 0.04	1.63 ± 0.06	1.24 ± 0.44	1.36 ± 0.52
ONE WAY ANOVA P VALUE			0.20	0.004*	0.55	0.001*
POSTHOC TEST	GROUP A	GROUP B	0.82	0.47	0.82	0.95
		GROUP C	0.18	0.004*	0.51	0.005*
	GROUP B	GROUP C	0.46	0.01*	0.86	0.002*

One way ANOVA followed by Bonferroni post hoc test

*p* < 0.05, statistically significant*; *p* > 0.05, not significant

At 6 weeks, a statistically significant difference was observed among the groups (*p* = 0.004, η² = 0.18), indicating a moderate effect size. The mean ± SD values showed a greater reduction in PI scores in Group A (Dente 91^®^) (1.24 ± 0.44; 95% CI: 1.04–1.44) and Group B (Enzycal 950™) (1.35 ± 0.20; 95% CI: 1.25–1.45) compared to Group C (control) (1.63 ± 0.06; 95% CI: 1.60–1.66), supporting the post hoc findings that Groups A (Dente 91^®^) and B (Enzycal 950™) performed significantly better than Group C (control). At 12 weeks, the mean ± SD scores for Group A (Dente 91^®^) (1.11 ± 0.39; 95% CI: 0.93–1.29), Group B (Enzycal 950™) (1.18 ± 0.18; 95% CI: 1.09–1.27), and Group C (control) (1.24 ± 0.44; 95% CI: 1.02–1.46) showed minimal differences, with no statistically significant variation among the groups (*p* = 0.55, η² = 0.01).

At 24 weeks, a statistically significant difference was again observed among the groups (*p* = 0.001, η² = 0.22), indicating a larger effect size. The mean ± SD PI scores demonstrated greater reduction in Group A (Dente 91^®^) (0.92 ± 0.32; 95% CI: 0.77–1.07) and Group B (Enzycal 950™) (0.88 ± 0.35; 95% CI: 0.71–1.05) compared to Group C (control) (1.36 ± 0.52; 95% CI: 1.10–1.62). Post hoc analysis further confirmed significant differences between Groups A (Dente 91^®^) and B (Enzycal 950™) versus Group C (control), reflecting reduction in plaque scores in former groups.

Considering, within-group comparisons, it was observed all experimental groups showed a highly significant difference across the specified time intervals i.e. 6,12 and 24th week (*p* = 0.0001) indicating effective intervention. However, between 6 and 12 weeks, neither group displayed any discernible differences. Group B (Enzycal 950™) demonstrated marked improvements between 12 and 24 weeks. In contrast, Group C (control) exhibited statistically significant reductions from baseline to the 12th and 24th weeks; however, no significant changes were observed between baseline and 6 weeks or between baseline and 12 weeks. Notable improvements in Group C (control) were evident during the interval from 6 to 12th weeks. However, the magnitude of PI score reduction was slightly lesser in Group C compared to the clinical outcome improvements relative to Groups A (Dente 91^®^) and B (Enzycal 950™).

The mean gingival (GI) scores at different study time intervals (baseline, 6 weeks, 12 weeks, and 24 weeks have been represented in Table 2. All participants exhibited moderate gingival inflammation at baseline, which improved by the 24th week across all groups. Intergroup comparison of GI values demonstrated no significant differences among the 3 experimental groups at baseline (ANOVA, *p* = 0.82, η² = 0.01) confirming comparable values. At 6 weeks, a substantial difference was observed between the experimental groups (*p* = 0.0002, η² = 0.34) with a large effect size, indicating a statistically significant changes in treatment outcomes. Among the experimental groups, the mean ± SD scores of Group A (Dente 91^®^), 1.28 ± 0.45 (95%CI: 1.07,1.49) and Group B (Enzycal 950™), 1.32 ± 0.20 (95%CI:1.22, 1.42) demonstrated comparatively reduced gingival scores compared to Group C (control), 1.67 ± 0.07 (95%CI:1.63, 1.71). At 12 weeks, the mean ± SD scores for Group A (Dente 91^®^), 1.06 ± 0.39 (95% CI: 0.88,1.24), Group B (Enzycal 950™) ,1.16 ± 0.17 (95% CI:1.08,1.24 ), and Group C (control), 1.22 ± 0.43 (95% CI:1.00,1.44 ) exhibited marginal differences, with no statistically significant variation among the groups (ANOVA, *p* = 0.38, η² = 0.03). At the final study time point i.e. 24th week, the comparisons between the experimental groups remained statistically significant (ANOVA, *p* = 0.002, η²=0.29) indicating consistent maintenance of treatment effects. The mean ± SD values for Group A (Dente 91^®^) (0.91 ± 0.32; 95% CI: 0.76–1.06) and Group B (Enzycal 950™) (0.87 ± 0.36; 95% CI: 0.69–1.05) were comparable, whereas higher values were observed in Group C (control) (1.31 ± 0.50; 95% CI: 1.06–1.56). These findings were further supported by post-hoc analysis, which revealed statistically significant differences between Groups A (Dente 91^®^) and B (Enzycal 950™) when compared to Group C (control), indicating superior gingival health outcomes in the former groups.

**Table 2:** Comparison of gingival index between study groups at different time intervals

		*N*	Baseline	6 weeks	12 weeks	24 weeks
GROUP A		16	1.83 ± 0.08	1.28 ± 0.45	1.06 ± 0.39	0.91 ± 0.32
GROUP B		18	1.81 ± 0.07	1.32 ± 0.20	1.16 ± 0.17	0.87 ± 0.36
GROUP C		15	1.83 ± 0.08	1.67 ± 0.07	1.22 ± 0.43	1.31 ± 0.50
ONE WAY ANOVA P VALUE			0.82	0.0002*	0.38	0.002*
POST HOC TEST	GROUP A	GROUP B	0.78	0.90	0.66	0.95
		GROUP C	0.90	0.0005*	0.36	0.011*
	GROUP B	GROUP C	0.76	0.001*	0.86	0.005*

One way ANOVA followed by Bonferroni post hoc test

*p* < 0.05, statistically significant*; *p* > 0.05, not significant

All experimental groups showed highly statistically significant decreases in gingival index scores within the groups from baseline to the 6,12 and 24 weeks of the study time intervals (*p* = 0.0001), indicating improved treatment outcomes. From baseline to all follow-up periods (baseline to 6 weeks, 12 weeks, and 24 weeks), Group A (Dente 91^®^) and B (Enzycal 950™) showed notable differences. As for Group C (control), they showed statistically significant reductions primarily from baseline to 12 and 24 weeks, and between 6 weeks and subsequent time points. However, the extent of GI score reduction was comparatively less with a slower rate of improvement relative to Groups A (Dente 91^®^) and B (Enzycal 950™).

The trends observed for PI and GI were consistent with bleeding on probing (BOP) scores, with all participants exhibiting moderate to severe BOP at baseline, which significantly decreased by the 24th week (Table 3). Inter group comparison at baseline demonstrated no statistically significant differences among the 3 experimental groups (ANOVA, *p* = 0.43; η² = 0.04) suggesting good baseline comparability between the groups. At 6 weeks, a statistically significant difference was observed between the experimental groups (ANOVA, *p* = 0.006; η² = 0.22) with a large effect size, signifying statistically effective differences in treatment. The mean ± SD scores of Group A (Dente 91^®^), 1.10 ± 0.39 (95%CI:0.92,1.28) and Group B (Enzycal 950™),1.19 ± 0.19 (95%CI:1.10,1.28) presented comparatively lesser bleeding scores compared to Group C (control), 1.44 ± 0.07 (95%CI: 1.40,1.48). At 12 weeks, intergroup comparison revealed no statistically significant difference with a small effect size (ANOVA, *p* = 0.40 η² = 0.03). The mean ± SD scores for Group A (Dente 91^®^), 0.98 ± 0.35 (95% CI:0.82,1.14), Group B (Enzycal 950™), 1.03 ± 0.26 (95% CI:0.90,1.16) and Group C (control), 1.13 ± 0.39 (95% CI: 0.93, 1.33) indicating comparable reductions in BOP scores at this interval. At 24th week time point, a substantially significant intergroup difference was observed with a larger effect size (ANOVA, *p* = 0.003, η²=0.27) specifying continued and relevant differences among the experimental groups. The mean ± SD values for Group A (Dente 91^®^) (0.85 ± 0.31; 95% CI: 0.71–0.99) and Group B (Enzycal 950™) (0.89 ± 0.26; 95% CI: 0.76–1.02) were comparable and lower than those observed in Group C (control) (1.24 ± 0.48; 95% CI: 1.00–1.48). These findings were further supported by post hoc analysis, which revealed statistically significant differences between Groups A (Dente 91^®^) and B (Enzycal 950™) when compared to Group C (control), indicating improved bleeding on probing outcomes in the former groups. Within-group comparison demonstrated greater statistically significant reductions in BOP scores over time in all groups (*p* = 0.0001). Both Group A (Dente 91^®^) and Group B (Enzycal 950™) demonstrated statistically significant reductions in bleeding scores from baseline to all follow-up time points (*p* = 0.0001), indicating effective improvement over time. In Group A (Dente 91^®^), the reduction was consistent and progressive across 6, 12, and 24 weeks, suggesting a steady treatment response throughout the study period. However, the differences between 6 and 12 weeks as well as between 12 and 24 weeks were not statistically significant suggesting that reduction in BOP occurred during the early phase and was subsequently consistent over the time period. Similarly, Group B (Enzycal 950™) also showed significant reductions at all study time intervals; however, an additional statistically significant improvement between 6 and 24 weeks indicated a continued treatment benefit with favourable long-term outcome. Overall, both interventions were effective in reducing bleeding scores, with Group B (Enzycal 950™) showing evidence of constant improvement over extended follow-up. Group C (control) exhibited statistically significant reductions from baseline to 12 and 24 weeks (*p* = 0.001); however, no significant change was observed between baseline and 6 weeks. Notable improvement was evident between 6 and 12 weeks, suggesting a delayed response compared to Groups A (Dente 91^®^) and B (Enzycal 950™).

**Table 3:** Comparison of bleeding on probing between study groups at different time intervals

		*N*	Baseline	6 weeks	12 weeks	24 weeks
GROUP A		16	1.50 ± 0.23	1.10 ± 0.39	0.98 ± 0.35	0.85 ± 0.31
GROUP B		18	1.48 ± 0.09	1.19 ± 0.19	1.03 ± 0.26	0.89 ± 0.26
GROUP C		15	1.51 ± 0.10	1.44 ± 0.07	1.13 ± 0.39	1.24 ± 0.48
ONE WAY ANOVA P VALUE			0.43	0.006*	0.40	0.003*
POSTHOC TEST	GROUP A	GROUP B	0.29	0.54	0.89	0.94
		GROUP C	0.75	0.006*	0.38	0.006*
	GROUP B	GROUP C	0.64	0.01*	0.65	0.01*

One way ANOVA followed by Bonferroni post hoc test

*p* < 0.05, statistically significant*; *p* > 0.05, not significant

**Fig. 2:**
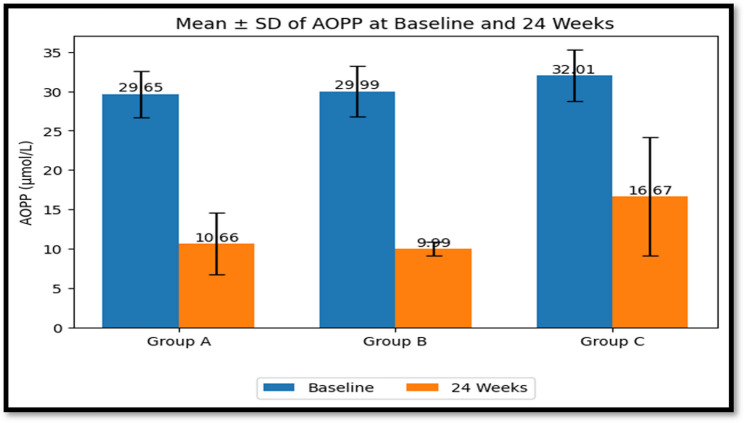
Graphical representation illustrating mean ± SD values of Advanced Oxidative Protein Product (AOPP) levels across the study groups at baseline and at 24 weeks

Based on the results obtained from the salivary biomarkers (Fig. 2), AOPP levels significantly decreased in all groups at the 24th week (*p* = 0.0001). Intergroup comparison at baseline showed, all 3 experimental groups had comparable mean ± SD scores Group A (Dente 91^®^): 29.65 ± 2.94( 95% CI: 28.29, 31.01), Group B (Enzycal 950™) : 29.99 ± 3.19 (95% CI: 28.43, 31.55), Group C (control): 32.01 ± 3.29 (95% CI: 30.35, 33.67), with no statistically significant difference observed between the groups ( ANOVA, *p* = 0.06, η² = 0.10), indicating moderate effect size and acceptable baseline comparability. Conversely, in 24 weeks, a statistically significant difference was observed among the groups (ANOVA, *p* = 0.0002, η² = 0.36), with a large effect size, indicating a substantial effect of the treatment. Group B (Enzycal 950™) exhibited the lowest mean ± SD score, 9.99 ± 0.85( 95% CI: 9.57,10.41), followed closely by Group A (Dente 91^®^), 10.66 ± 3.91 ( 95% CI: 8.85,12.47), while Group C (control) showed comparatively higher mean ± SD values, 16.67 ± 7.56 (95% CI: 12.85,20.49), suggesting reduced effectiveness in Group C (control). Considering, within group analysis, a statistically significant reduction was noticed from baseline to 24 weeks in both Group A (Dente 91^®^) and Group B (Enzycal 950™) (ANOVA, *p* = 0.0001), indicating enhanced treatment effect in these groups. In contrast, Group C (control) did not demonstrate a statistically significant reduction over time (ANOVA, *p* = 0.09), despite a decrease in mean values, suggesting comparatively lower efficacy of the intervention.

**Fig. 3:**
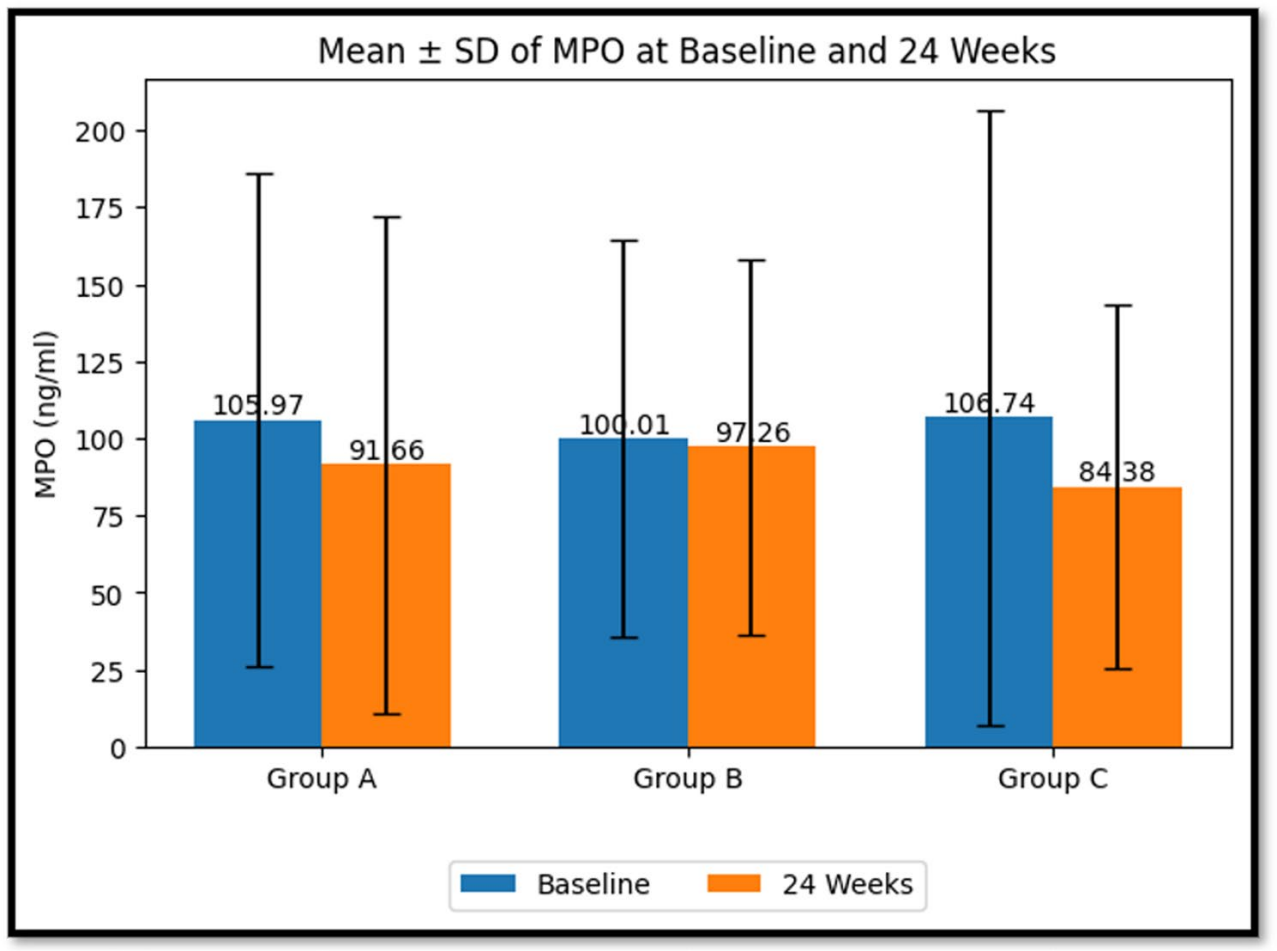
Graphical representation illustrating mean ± SD values of Myeloperoxidase (MPO)levels across the study groups at baseline and at 24 weeks

Figure 3 described the results of MPO salivary biomarker. At baseline, no statistically significant intergroup difference was observed among the three experimental groups (Kruskal–Wallis, *p* = 0.86), with a very small effect size (η² = 0.01). The mean ± SD levels for Group A (Dente 91^®^) ,105.97 ± 79.91 (95% CI: 69.05,142.89), Group B (Enzycal 950™), 100.01 ± 64.47 (95% CI: 68.33–131.69) and 106.74 ± 99.79 (95% CI: 56.26,157.22) for Group C (control), indicating comparable baseline levels among the groups. Likewise, in 24 weeks, the intergroup comparison again revealed no statistically significant difference (Kruskal–Wallis, *p* = 0.76), with a small effect size (η² = 0.02). The mean ± SD values recorded were 91.66 ± 80.61(95% CI: 54.43, 128.89) for Group A (Dente 91^®^), 97.26 ± 61.08 (95% CI: 67.35, 127.17) for Group B (Enzycal 950™), and 84.38 ± 59.14 (95% CI: 54.50, 114.26) for Group C (control). Within-group analysis also demonstrated no statistically significant changes from baseline to 24 weeks in Group A (Dente 91^®^) (*p* = 0.59), Group B (Enzycal 950™) (*p* = 0.89), or Group C (control) (*p* = 0.76), indicating that the MPO levels remained relatively invariable across the study period.

**Fig. 4:**
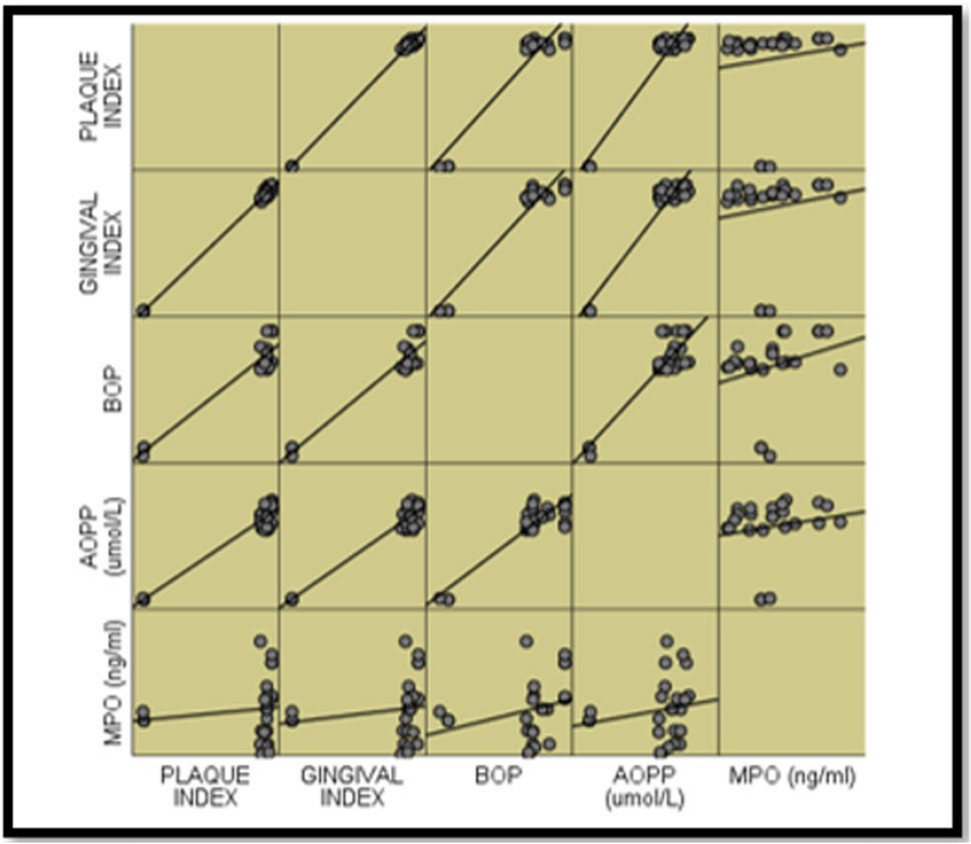
Clustered graph depicting Pearson’s correlation between clinical and biochemical parameters across the study groups

The Pearson’s correlation(r) analysis (Fig. 4) was performed to identify linear relationships between the variables recorded in the present study. The correlation matrix presented distinct patterns of associations among the clinical and biochemical parameters across the three experimental groups.

Group A (Dente 91^®^) exhibited a statistically significant positive correlation between PI and GI scores (*r* = 0.996, *p* < 0.001), PI and bleeding on probing (BOP) (*r* = 0.917, *p* < 0.001), and PI and advanced oxidation protein products (AOPP) (*r* = 0.941, *p* < 0.001). Similarly, GI scores demonstrated strong positive correlations with BOP (*r* = 0.933, *p* < 0.001) and AOPP (*r* = 0.939, *p* < 0.001), while BOP also showed a significant positive correlation with AOPP (*r* = 0.876, *p* < 0.001). No significant correlations were observed between myeloperoxidase (MPO) and any of the assessed parameters (*p* > 0.05). Group B (Enzycal 950™), displayed moderate positive correlations between GI scores and BOP (*r* = 0.487, *p* = 0.040) as well as GI scores and AOPP (*r* = 0.505, *p* = 0.033), which were statistically significant. However, correlations between PI and other parameters (GI, BOP, AOPP, and MPO) were weak and not statistically significant (*p* > 0.05). Considering, Group C (control), most of the correlations were weak. However, a statistically significant moderate negative correlation was observed between gingival scores and AOPP (*r* = -0.470, *p* = 0.049), indicating an inverse relationship between the two variables. All other associations, including those involving plaque, BOP, and MPO, were not statistically significant (*p* > 0.05). Overall, among the experimental groups, A (Dente 91^®^) exhibited strong and reliable positive correlations among clinical and oxidative stress biomarkers, whereas Groups B (Enzycal 950™) and C (control) demonstrated weaker and less consistent associations, with limited statistical significance.

## Discussion

Orthodontic appliances complicate oral hygiene by fostering plaque accumulation, thereby increasing the risk of gingival inflammation [25, 26]. Dentifrices, particularly those incorporating enzymes or proteins, have demonstrated promise in enhancing oral defence mechanisms. Studies in orthodontic cohorts have shown that enzyme-based formulations improve microbial balance and boost salivary defence systems. A randomized controlled trial reported a shift toward a periodontal health-associated microbiome after 14 weeks of enzymatic dentifrice use [27], and similar research found elevated enzyme levels after one month of use, supporting their immunomodulatory potential [28]. 

The present randomized controlled clinical trial evaluated the anti-plaque efficacy of two enzyme-containing dentifrices, Dente 91^®^ and Enzycal 950™ compared to a conventional fluoride dentifrice, Colgate^®^ Strong teeth in orthodontic patients diagnosed with chronic gingivitis over a 24-week follow-up period. The null hypothesis was rejected, as both tested dentifrices showed statistically significant favourable anti-plaque efficacy and improved gingival outcomes compared to the control dentifrice at the 24-week follow-up.

Clinical indices including plaque index (PI), gingival index (GI), and bleeding on probing (BOP) were used to assess oral hygiene status. The 24-week duration was chosen based on prior findings that dentifrice effects are best observed over six months [29, 30].

The clinical improvements observed over the 24 -week period may be attributed to reinforced oral hygiene instructions and participant compliance. However, the lack of statistically significant changes at certain time intervals, both intergroup and intragroup, may be explained by inconsistent dentifrice usage or inflammation caused by ongoing orthodontic treatment. Similar trends have been observed in earlier trials, where plateauing effects were noted beyond early usage phases [15, 26, 31].

Notably, Group A (Dente 91^®^) and Group B (Enzycal 950™) demonstrated significantly greater reductions in PI, GI, and BOP compared to the control (Colgate^®^ Strong) group particularly at the 6th and 24th weeks. These findings demonstrated improvement of the enzyme-based dentifrices; however, the clinical relevance of these differences needs to be considered cautiously. The observed reduction in plaque accumulation and gingival inflammation suggests improved oral hygiene status with lower risk of gingivitis and better periodontal health in orthodontic patients. Furthermore, the concomitant reduction in BOP indicates a decrease in gingival inflammation. However, the clinical significance of these findings should be considered based on baseline scores, effect size and inter-individual variability as statistical significance does not always translate into substantial clinical benefit. Overall, the results of the present study suggest that improvements are both statistically and clinically relevant, particularly in context to plaque control and gingival health challenges associated with fixed orthodontic therapy. The observed improvements with the enzymatic dentifrices may be attributed to their unique bioactive components available in the dentifrice. Dente 91^®^ combines nanohydroxyapatite and lactoferrin, both known for its antimicrobial and anti-biofilm properties [32, 33]. Lactoferrin deprives microbes of iron and alters membrane permeability [9], while nanohydroxyapatite disrupts bacterial membranes and releases ions that neutralize acidogenic environments. Likewise, Enzycal 950™, containing amyloglucosidase, glucose oxidase, and lactoperoxidase, exerts antibacterial effects by generating hydrogen peroxide and hypothiocyanite, which impair bacterial metabolism [10, 28]. Previous studies corroborate these findings, with significant improvements observed in clinical indices after the use of enzymatic dentifrices [15, 26, 31]. The findings align with literature demonstrating the antimicrobial and immunological benefits of enzyme-based formulations. Hatti et al. observed that enzyme-containing dentifrices prevent biofilm formation [34], while Hannig et al. reported an enhancement in their antimicrobial activity [35]. Previous studies have reported that salivary protein-enriched dentifrices can restore antimicrobial function [36], disrupt bacterial membrane integrity [37], and substantially reduce gingival inflammation and bleeding on probing over one year [8].

Oxidative stress levels, assessed via salivary AOPP and MPO markers, offered additional insight in this study. Groups A (Dente 91^®^) and B (Enzycal 950™) exhibited significant reductions in AOPP by week 24, while Group C showed non-significant changes. Lactoferrin within the enzymatic dentifrices may have contributed by scavenging free radicals and suppressing pro-inflammatory cytokines [36, 38]. The enzyme system in Enzycal 950™ may have similarly reduced microbial-induced oxidative stress [27, 39, 40]. These results align with López-Mateos et al., who noted increased AOPP levels during initial orthodontic therapy, likely due to appliance-induced inflammation [11].

Although MPO levels showed reduction in all groups, the changes were not statistically significant, possibly due to persistent subclinical gingival inflammation or the nature of enzymatic dentifrices, which may act by modulating host response rather than a neutrophil-derived activity. Prior evidence suggests that MPO levels can remain elevated even after clinical resolution of inflammation resolves [41]. Similar findings were noted by López-Mateos et al., with unchanged MPO levels despite different orthodontic interventions [11]. Regarding the correlation between clinical parameters and oxidative stress biomarkers, a significant association was observed between AOPP and clinical indices, indicating a link between gingival inflammation and oxidative damage. These findings agree with Maciejczyk et al., who reported positive correlations between AOPP, PI, and GI [42]. Conversely, Babasova et al. found no such correlation in their study comparing periodontitis and healthy subjects [43]. No significant correlation was found between MPO and clinical indices in the present study, indicating that MPO may be a less specific biomarker in orthodontic settings. However, these findings are contrary to those reported by Dagar et al. and Smith et al., who observed strong MPO associations with gingivitis parameters [41, 44]. Previous trials have integrated clinical parameters with oxidative stress markers in orthodontic populations using enzyme-based dentifrices. The presents study adds to this evidence by providing comprehensive clinical and biochemical data, highlighting the sustained effects of these enzyme-based formulation. The present study has several limitations that should be considered when interpreting the findings. The relatively small sample size may limit the generalizability of the results to a broader population undergoing orthodontic therapy. Although blinding was maintained, involvement of a single investigator in both treatment delivery and outcome assessment may introduce a potential risk of performance or detection bias. Inconsistent brushing compliance may act as a confounding factor, resulting in changes to both the extent and direction of the observed effects. Variations in individual adherence during the study period could have influenced the clinical outcomes across all groups, thereby making it difficult to accurately determine the true efficacy of the intervention. Additionally, the lack of microbiological assessment in this study limits the understanding of the underlying mechanisms of action of the tested dentifrices.

## Conclusion

The findings observed in this randomized controlled clinical trial performed on patients undergoing fixed orthodontic therapy suggest that these enzyme-based dentifrices, were associated with improvements in the plaque and gingival parameters at the specific study time intervals compared to conventional dentifrice. Considering the limitations of this clinical study, the proposed biomimetic mechanism was not directly evaluated, and the absence of microbiological data and specific effect size estimation may confine the interpretation of results and clinical relevance of these findings. As the study assessed only gingival outcomes, the results cannot be generalized to other oral health parameters, including caries. Furthermore, clinical studies which include larger sample size, comprehensive microbiological and clinical assessments, extended recall intervals are required to determine their long-term clinical effectiveness and benefits.

## Data Availability

All data are available whenever requested from the corresponding author of the manuscript.
